# Defining and supporting a professional role for pharmacists associated with traditional and complementary medicines: a cross-country survey of pharmacists

**DOI:** 10.3389/fphar.2023.1215475

**Published:** 2023-08-16

**Authors:** Joanna E. Harnett, Shane P. Desselle, Marcília Baticy Fernandes, Dongning Yao, Darko Modun, Souheil Hallit, Mariam Dabbous, Mohd Shahezwan Abd Wahab, Afonso Miguel Cavaco, Maria Magalhães, Erwin Martinez Faller, Jennifer M. Flores, Jacklyn Risia D. San Gabriel, Noordin Othman, Puree Anantachoti, Tatta Sriboonruang, Wanna Sriviriyanupap, Faris Alnezary, Yaser Alahmadi, Saad Bakur Fallatah, Haifa Abdulrahman Fadil, Carolina Oi Lam Ung

**Affiliations:** ^1^ School of Pharmacy, Faculty of Medicine and Health, The University of Sydney, Sydney, NSW, Australia; ^2^ Touro University California College of Pharmacy, Vallejo, CA, United States; ^3^ Departamento de Ciências da Saúde, Ambiente e Tecnologias, Universidade de Santiago, Assomada, Cabo Verde; ^4^ School of Pharmacy, Nanjing Medical University, Nanjing, China; ^5^ Department of Pharmacy, University of Split School of Medicine, Split, Croatia; ^6^ School of Medicine and Medical Sciences, Holy Spirit University of Kaslik, Jounieh, Lebanon; ^7^ Applied Science Research Center, Applied Science Private University, Amman, Jordan; ^8^ Research Department, Psychiatric Hospital of the Cross, Jal Eddib, Lebanon; ^9^ School of Pharmacy, Lebanese International University, Beirut, Lebanon; ^10^ Faculty of Pharmacy, Universiti Teknologi MARA (UiTM) Cawangan Selangor, Selangor, Malaysia; ^11^ Non-Destructive Biomedical and Pharmaceutical Research Centre, Smart Manufacturing Research Institute, Universiti Teknologi MARA (UiTM) Cawangan Selangor, Selangor, Malaysia; ^12^ Universidade de Lisboa, Faculdade de Farmácia, Departamento de Farmácia Farmacologia e Tecnologias em Saúde, Lisboa, Portugal; ^13^ Pharmacy Department, School of Allied Health Sciences, San Pedro College, Davao City, Philippines; ^14^ Institute of Pharmacy, University of Makati, Makati City, Philippines; ^15^ School of Pharmacy, Emilio Aguinaldo College, Cavite City, Philippines; ^16^ Clinical and Hospital Pharmacy Department, College of Pharmacy, Taibah University, Al-Madinah Al-Munawwarah, Saudi Arabia; ^17^ School of Pharmacy, Management and Science University, University Drive, Off Persiaran Olahraga, Selangor, Malaysia; ^18^ Faculty of Pharmaceutical Sciences, Chulalongkorn University, Bangkok, Thailand; ^19^ State Key Laboratory of Quality Research in Chinese Medicine, Institute of Chinese Medical Sciences, University of Macau, Taipa, Macao, China

**Keywords:** public health, health policy, traditional and complementary medicines, pharmacist, pharmacy practice

## Abstract

**Introduction:** An estimated 80% of the world’s population use traditional and complementary medicine (T&CM) products as part of their healthcare, with many accessed through pharmacy. This cross-cultural study posed a set of professional practice responsibilities and actions to pharmacists related to T&CM products, with a view toward developing consensus, safeguarding, and promoting the health of the public.

**Methods**: Data were collected from 2,810 pharmacists across nine countries during 2022 via a cross-sectional online survey reported in accordance with the guidelines of STrengthening the Reporting of OBservational studies in Epidemiology (STROBE) and the Checklist for Reporting Results of Internet E-Surveys (CHERRIES).

**Results**: Of the 2,810 participants from nine countries, 2,341 completed all sections of the survey. Of these, most agreed (69%) that T&CM product use was common in the community they served, but most did not have adequate training to support consumer needs. Over 75% acknowledged that there were known and unknown safety risks associated with T&CM use. Of 18 professional responsibilities posed, 92% agreed that pharmacists should be able to inform consumers about potential risks, including T&CM side effects and drug–herb interactions. The provision of accurate scientific information on the effectiveness of T&CM products, skills to guide consumers in making informed decisions, and communication with other healthcare professionals to support appropriate and safe T&CM product use were all ranked with high levels of agreement. In order to effectively fulfill these responsibilities, pharmacists agreed that regulatory reforms, development of T&CM education and training, and access to quality products supported by high-quality evidence were needed.

**Conclusion:** General agreement from across nine countries on eighteen professional responsibilities and several stakeholder actions serve as a foundation for the discussion and development of international T&CM guidelines for pharmacists.

## 1 Introduction

Implementation of the Traditional Medicine Policy 2014–2023 of the World Health Organization (WHO) in “promoting the safe and effective use of Traditional and Complementary Medicine (T&CM) through the regulation, research and integration of T&CM products, practices and practitioners into the health systems as appropriate” continues to unfold across the world (The World Health Organisation, 2014). The terms used when referring to T&CM products vary across regions depending on the regulatory frameworks for medicines and food. The terms “traditional medicine” and “health supplements” are used in China and parts of South and Southeast Asia, the term “complementary medicines” (CMs) is used in Australia, and the term “dietary supplement” is used in the United States (US) (The World Health Organisation, 2019). For this study, the term T&CM products will be employed to refer to products containing vitamins, minerals, herbs, essential oils, and homeopathic and flower essence preparations, regardless of the regulatory status within a country. Throughout the world, pharmacists encounter T&CM product use in their day-to-day practice through direct provision of products or indirectly while caring for patients who use them. This aligns with the WHO statement related to the mission of pharmacy practice: “to provide medications and other healthcare products and services, and to help people and society to make the best use of them” (The World Health Organization, 2011).

An estimated 80% of the world population use T&CM products as part of their healthcare (The World Health Organisation, 2014). Such prevalent use is associated with risks and benefits and has implications for public health. As T&CM products are generally considered low risk by most regulators, they are directly marketed to consumers and predominantly self-selected in retail outlets, including pharmacies. Pharmacists’ contribution to public health through the professional stewardship of prescription and over-the-counter medicine use is well established. However, there are currently no data comparing the practicalities and formal processes required to engage pharmacists in caring for people who use T&CM products. Over the last 20 years, there have been deliberations about advancing and formalizing pharmacists’ professional responsibilities in promoting the safe and appropriate use of T&CM products (The World Health Organisation, 2019). These discussions have resulted in messaging by professional organizations recommending pharmacists adopt professional practice behaviors associated with T&CM products. The evidence suggests that pharmacists acknowledge that incorporating stewardship of T&CM product use into pharmaceutical services is needed and of value (Ung et al., 2017a; Harnett et al., 2019; Yao et al., 2020). However, the evidence suggests that pharmacists are not proactively engaging with the public about their T&CM product use and overall are underperforming in this area (Lee et al., 2021). These concerning observations have been reported in countries with a high prevalence of T&CM product use: the United States (Harnett et al., 2019), Australia (Lee et al., 2021), China (Yao et al., 2020), and some other countries (Ung et al., 2017b).

A recent review involving data analysis from 30 countries, exploring the interface between T&CM product use and the pharmacist role, reported that research advocating and strategizing change in this area has grown in the last 5 years (Ung et al., 2017b). Moving beyond soft recommendations for professional practice behaviors associated with T&CM products to establishing professional standards is considered important (Harnett et al., 2019). Therefore, there is a need to listen to the pharmacy professions to determine an agreed set of reasonable professional practice responsibilities and the competencies required to fulfill them. Competency standards are foundational in informing the development of pharmacists’ education and associated learning outcomes, thus equipping pharmacists to meet such professional practice requirements.

The need for pharmacists to step up their role with regard to T&CM products as an extension of their professional duties has been repeatedly discussed. More recently, a bioethical framework has been proposed to guide pharmacists’ responsibilities associated with the sale of T&CM products (Popattia and La Caze, 2021). This framework concurs that there is a need for standardizing a set of professional practice responsibilities that center around pharmacists providing evidence-based information and consultations when purchasing products (Popattia and La Caze, 2021). Meanwhile, public health needs remain wanted with obvious gaps between these recommendations and the T&CM pharmaceutical care being delivered (Lee et al., 2021; Ng et al., 2021). This disconnection is indirectly supported by the fact that in most countries, the legal and regulatory frameworks associated with T&CM are not clearly defined (Ung et al., 2017a).

Given the nuances, cultural context, history of traditional medicines use, and the political and commercial interests associated with complementary medicines, any changes to legal frameworks and associated practice standards of a profession associated with T&CM products are complex. Therefore, both the voices of pharmacists and the public should be present in conversations about professional practice responsibilities and any legal requirements that may ensue. Meeting public health needs in day-to-day practices requires considering the diverse communities that use T&CM products and how these products are integrated (or not) into different healthcare systems and into pharmacist education across the world. Other relevant stakeholders in these conversations include pharmacy educators, professional pharmacist organizations, universities, the government, and pharmacy business owners (Ung et al., 2018). This requires a considered effort from pharmacists and their representative organizations toward such a consensus that translates the last two decades of research into implementable global practice behaviors that support public health and safety.

The first step toward greater standardization, development, and implementation of practice standards and ultimately guidelines is to garner up-to-date information regarding pharmacists’ beliefs about T&CM-related responsibilities, education, and their current practice behaviors from various locations around the world that vary by income status and health and education systems. Identifying areas of agreement and potential discrepancies across these countries will assist in developing a set of realistic professional practice responsibilities informed by real-world practice. These can be used to inform the development of education, competency, and practice standards.

The overarching aim of this cross-country study was to identify an agreed set of professional pharmacy practice responsibilities that support pharmacists’ contribution to ensuring the quality and safe use of T&CM products and promote public health. The objectives were 1) to develop, pilot, and disseminate a cross-country e-survey through a cross-country collaboration with professional associations and representative organizations and 2) to provide a cross-country perspective of pharmacists’ opinions about their professional practice responsibilities associated with T&CM products and, importantly, the steps and support required to formalize a professional practice role. The findings of this study will be used to inform the next research stage in developing a consensus on professional pharmacy practice responsibilities associated with T&CM products.

## 2 Methods

### 2.1 Study design

A cross-sectional online survey with convenience sampling was conducted across nine countries: Carbo Verde, China, Croatia, Lebanon, Malaysia, Philippines, Portugal, Saudi Arabia, and Thailand. The full study protocol is reported elsewhere (Harnett et al., 2022) in accordance with the guidelines of STrengthening the Reporting of OBservational studies in Epidemiology (STROBE) (Von Elm et al., 2007) and the Checklist for Reporting Results of Internet E-Surveys (CHERRIES) (Eysenbach, 2004). This cross-country collaboration was led by researchers based at the Institute of Chinese Medical Sciences, University of Macau, and The University of Sydney School of Pharmacy, Faculty of Medicine and Health in New South Wales, Australia.

To select the participating countries, the lead researchers COUL and JH scoped Medline to identify collaborators who had published research exploring the interface between pharmacy practice and T&CM within the last 5 years. Collaborators representing countries across the three income categories (low-, middle-, and high-income countries) and across the six WHO regions were identified to provide a diverse socio-economic and demographic representation. Researchers from the African Region (Cabo Verde), the Southeast Asian Region (China, Malaysia, Philippines, and Thailand), the European Region (Croatia, Portugal, and Lebanon), the Eastern Mediterranean Region (Saudi Arabia), the Western Pacific (Australia), and Region of Americas (the United States and Brazil) and representatives of low-, middle-, and high-income countries were contacted. After a series of email communications with the researchers identified, an online meeting was conducted in November 2021 to discuss the project protocol and ensure that the aims and objectives of the project were clear. The survey was disseminated across the nine countries between April and June 2022. The recruitment strategy used by individual countries included the survey link sent by pharmacist’s professional associations and special interest groups *via* emails or apps. The United States, Australian, and Brazilian collaborators were not included in the final study due to recruitment challenges imposed by the global pandemic.

Ethics approval to conduct the study across the nine countries involved was obtained from the University of Macau (approval number SSHRE21-APP068-ICMS-01). The local collaborators in each country were responsible for meeting any additional ethical requirements relevant to their country and for engaging with relevant pharmacy organizations to recruit eligible participants. Therefore, additional ethics applications and approval were obtained to conduct the study in Thailand (Chulalongkorn University COA No. 087/65). All other countries conducted their research in accordance with the approval of the University of Macau cited above. Participation in the survey was voluntary and completed anonymously.

### 2.2 Study population

The target population within the nine countries were pharmacists, who were adults of any age and gender and located in the broader pharmacy-related settings of community, hospital, consultancy, industry, academia, and regulatory bodies.

Sample size: the sample size calculation varied based on the size of the pharmacy workforce. Other factors considered when calculating the sample size were accessibility to the lists of registered pharmacists within the respective country. The minimum sample size for each country was targeted to achieve a 95% confidence interval, with a margin of error of 5% for the survey results. The minimum sample size was unmet in all countries (see Supplementary File S1).

### 2.3 Survey instrument development

Survey questions were informed by the results of two systematic reviews published in 2017^7^ and 2022^8^ that explored the professional role and responsibilities of pharmacists in relation to T&CM products. The survey questions were developed and first tested in English. The survey was constructed using the online survey platform Survey Monkey. The processes for developing and testing the survey are outlined in the study protocol (Harnett et al., 2022). When translation from English to another language was required for dissemination in non-English speaking countries, the collaborator located in that country organized the translation of the survey instrument and further testing for face validity. Additionally, when a survey was translated from English, a third step was conducted involving back translation, whereby survey items were translated back to English to ensure that the intent of the questions was not lost in translation. Testing of the instrument in all countries was conducted before recruitment, and each country had a separate link to the survey for the purpose of data collection. The survey was translated into Croatian (for Croatia), Portuguese (for Portugal and Carbo Verde), Chinese (for China), and Thai (for Thailand) and disseminated in English for all the other countries.

### 2.4 The survey instrument and items

A total of 43 items focused on pharmacists and T&CM products formed the survey (see S2) (Harnett et al., 2022). A question confirming consent to participate was embedded following a brief participant information statement outlining the research and the purpose of the study. The items were categorized into five sections: Section 1, demographics; Section 2, pharmacists’ perceptions about T&CM; Section 3, pharmacists’ opinions about bioethical responsibilities; Section 4, pharmacists’ opinions about practice responsibilities; and Section 5, the infrastructure and support required. The survey took 9–11 min to complete.

### 2.5 Data analysis

As outlined in the study protocol (Harnett et al., 2022), the anonymous meta-data collected from the nine countries were stored in Survey Monkey before exporting to Excel and the Statistical Package for Social Sciences (SPSS) version 27 software for Windows for analysis. Section 1 of the survey (i.e., participants’ demographic data) was analyzed descriptively. Data obtained from Likert scales of agreement in Section 2, pharmacists’ perceptions about T&CM; Section 3, pharmacists’ opinions about bioethical responsibilities; Section 4, pharmacists’ opinions about practice responsibilities; and Section 5, the infrastructure and support required were analyzed as nominal/categorical data—number and percentage of participants responding per level of agreement. Given the sample size variations per country and the similarity in levels of agreement across countries for most statements, multiple linear regression was not conducted per the published protocol, and the data were not analyzed by construct for factor analysis.

## 3 Results

### 3.1 Demographic characteristics

As presented in Table 1, 2,810 pharmacists representing nine countries participated in the survey representing three low-middle-income countries (LMIC), three upper middle-income countries (UMIC), and three high-income countries (HIC): Carbo Verde, *n* = 76; China, *n* = 540; Croatia, *n* = 316; Lebanon, *n* = 1,339; Malaysia, *n* = 497; Philippines, *n* = 276; Portugal, *n* = 136; Saudi Arabia, *n* = 156; and Thailand, *n* = 261. Among the 2,810 participants who responded, 2,341 pharmacists completed all survey questions, giving an overall completion rate of 83% (ranging from 72.4% in Saudi Arabia to 100% in China). The mean age of the 2,341 survey participants was 38.0 ± 10.3 years, and the majority [1,654 (71%)] were females. Most participants [1,418 (61%)] worked in community pharmacies, followed by hospital pharmacies [454 (19%)]. The majority [1,757 (75%)] were employed, and 20% were business owners. Less than half of the survey participants [876 or 37% (ranging from 4% in Portugal to 63% in Lebanon)] reported receiving comprehensive training in their undergraduate pharmacy training. A further 1,117 participants (48%) reported the inclusion of T&CM education in their undergraduate training but believed it was not comprehensive enough, and 348 (15%) had received no T&CM education at all.

**TABLE 1:** Demographic characteristics.

Participant characteristics			Participating countries (number of participants)									Total (*n* = 2,341)
			Carbo Verde (*n* = 61)	China (*n* = 540)	Croatia (*n* = 203)	Lebanon (*n* = 627)	Malaysia (*n* = 379)	Philippines (*n* = 196)	Portugal (*n* = 82)	Saudi Arabia (*n* = 71)	Thailand (*n* = 182)	
Country income category (World Bank)			LMIC	U MIC	HIC	LMIC	UMIC	LMIC	HIC	HIC	UMIC	
Gender	Male	*n* (%)	11 (18)	161 (30)	20 (10)	233 (37)	105 (28)	41 (21)	6 (7)	43 (61)	58 (32)	678 (29)
	Female		50 (82)	379 (70)	182 (90)	394 (63)	273 (72)	155 (79)	76 (93)	28 (39)	117 (64)	1,654 (71)
	Non-binary/third gender		0 (0)	0 (0)	1 (0.x)	0 (0)	0 (0)	0 (0)	0 (0)	0 (0)	6 (3)	7 (0.x)
	Prefer not to say		0 (0)	0 (0)	0 (0)	0 (0)	1 (0.x)	0 (0)	0 (0)	0 (0.0)	1 (1)	2 (0.x)
Age	Mean ± SD		37.9 ± 8.6	41.2 ± 8.1	37.9 ± 10.3	34.8 ± 10.3	33.7 + 6.6	42.4 ± 14.2	44.9 ± 10.1	31.3 ± 6.0	43.8 ± 9.8	38.0 ± 10.3
Practice area (more than 1 option can be chosen)	Community pharmacist	*n* (%)	37 (61)	373 (69)	174 (86%)	528 (84)	80 (21)	83 (42)	75 (91)	18 (25)	50 (27)	1,418 (61)
	Hospital pharmacist		12 (20)	55 (10)	5 (2)	42 (7)	193 (51)	39 (20)	1 (1)	39 (55)	68 (37)	454 (19)
	Clinical pharmacist (consultant)		10 (16)	0 (0)	2 (1)	107 (17)	13 (3)	6 (3)	6 (7)	3 (4)	4 (2)	151 (6)
	Pharmaceutical industry		7 (11)	220 (41)	10 (5)	95 (15)	5 (1)	25 (13)	1 (1)	6 (8)	19 (10)	388 (17)
	Academic		13 (21)	12 (2)	9 (4)	71 (11)	31 (8)	42 (21)	0 (0)	8 (11)	13 (7)	199 (9)
	Pharmacist (regulatory body)		0 (0)	9 (2)	2 (1)	0 (0)	24 (6)	12 (6)	0 (0)	2 (3)	31 (17)	80 (3)
	Others		11 (18)	12 (2)	6 (3)	79 (13)	51 (13)	22 (11)	5 (6)	3 (4)	17 (9)	206 (9)
Employment status	Employed as a pharmacist	*n* (%)	51 (84)	461 (85)	187 (92)	290 (46)	352 (93)	148 (76)	56 (68)	67 (94)	145 (80)	1,757 (75)
	Self-employed pharmacist		7 (11)	47 (9)	11 (5)	285 (45)	23 (6)	38 (19)	22 (27)	1 (1)	26 (14)	460 (20)
	Not employed		3 (5)	19 (4)	4 (2)	50 (8)	4 (1)	3 (2)	2 (2)	3 (4)	3 (2)	91 (4)
	Retired		0 (0)	13 (2)	1 (0)	2 (0)	0 (0)	7 (4)	2 (2)	0 (0)	8 (4)	33 (1)
Inclusion of T&CM in undergraduate pharmacy education and training	Yes (comprehensive)	*n* (%)	9 (15)	199 (37)	24 (12)	395 (63)	88 (23)	74 (38)	3 (4)	28 (39)	56 (31)	876 (37)
	Yes (but not comprehensive)		29 (48)	281 (52)	132 (65)	174 (28)	243 (64)	84 (43)	25 (30)	33 (46)	116 (64)	1,117 (48)
	No		23 (38)	60 (11)	47 (23)	58 (9)	48 (13)	38 (19)	54 (66)	10 (14)	10 (5)	348 (15)

### 3.2 Pharmacists’ exposure to and perceptions of traditional and complementary medicines

Most pharmacists agreed or strongly agreed [1,611 or 69% (ranging from 55% in Portugal to 84% in China)] that T&CM product use was common in the community they served and that there was evidence to support the efficacy of some products in specific conditions [1,655 or 71% (ranging from 45% in Thailand to 94% in China)]. More than three-quarters of the pharmacists surveyed [1,925 or 78% (ranging from 70% in Lebanon to 84% in Cabo Verde)] acknowledged that there were known and unknown safety risks associated with T&CM use.

As shown in Table 2, more than 4 out of 5 participants [1,937 or 83% (ranging from 68% in Saudi Arabia to 95% in China] agreed or strongly agreed that T&CM product use should be included in their scope of practice, whereas a small minority [123 or 5% (ranging from 1% in Philippines to 11% in Portugal)] disagreed or strongly disagreed to considering the inclusion of T&CM in their scope of practice. Regarding the types of T&CM products that should be included in pharmacy practice, as shown in Table 2, a significant proportion of participants favored the inclusion of specific products as follows: 2,065 participants (or 88%) (ranging from 79% in Saudi Arabia to 95% in Croatia), herbal products; 1,944 (or 85%) (ranging from 72% in Croatia to 91% in Lebanon), vitamin and mineral supplements; 1,855 (or 79%) (ranging from 55% in Saudi Arabia to 89% in Portugal), nutritional products (other than vitamins and minerals); and 1,841 (or 79%) (ranging from 58% in Saudi Arabia to 93% in Portugal), pro- and prebiotics. Only 896 participants (or 38%) (ranging from 0% in China to 72% in Thailand) believed that homeopathic products should be included in pharmacists’ scope of practice.

**TABLE 2:** Pharmacists’ exposure to and perceptions about T&CM products.

Level of agreement		Participating countries									Total (*n* = 2,341)
		Carbo Verde (*n* = 61)	China (*n* = 540)	Croatia (*n* = 203)	Lebanon (*n* = 627)	Malaysia (*n* = 379)	Philippines (*n* = 196)	Portugal (*n* = 82)	Saudi Arabia (*n* = 71)	Thailand (*n* = 182)	
Survey statement 1: “T&CM product use is common in the community I serve”											
Strongly disagree	*n* (%)	1 (2)	12 (2)	8 (4)	30 (5)	22 (6)	7 (4)	6 (7)	3 (4)	11 (6)	100 (4)
Disagree		2 (3)	51 (9)	21 (10)	50 (8)	33 (9)	18 (9)	12 (15)	6 (8)	21 (12)	214 (9)
Neither agree nor disagree		10 (16)	23 (4)	54 (27)	146 (23)	72 (19)	37 (19)	19 (23)	16 (23)	39 (21)	416 (18)
Agree		23 (38)	362 (67)	70 (34)	181 (29)	108 (28)	59 (30)	27 (33)	19 (27)	42 (23)	891 (38)
Strongly agree		25 (41)	92 (17)	50 (25)	220 (35)	144 (38)	75 (38)	18 (22)	27 (38)	69 (38)	720 (31)
Survey statement 2: “There is evidence to support the efficacy of some T&CM products in specific conditions”											
Strongly disagree	*n* (%)	2 (3)	1 (0.x)	3 (1)	11 (2)	10 (3)	3 (2)	4 (5)	4 (6)	16 (9)	54 (2)
Disagree		7 (11)	24 (4)	14 (7)	33 (5)	27 (7)	12 (6)	11 (13)	7 (10)	32 (18)	167 (7)
Neither agree nor disagree		8 (13)	8 (1)	32 (16)	163 (26)	119 (31)	46 (23)	14 (17)	22 (31)	53 (29)	465 (20)
Agree		24 (39)	379 (70)	69 (34)	211 (34)	139 (37)	65 (33)	31 (38)	23 (32)	45 (25)	986 (42)
Strongly agree		20 (33)	128 (24)	85 (42)	209 (33)	84 (22)	70 (36)	22 (27)	15 (21)	36 (20)	669 (29)
Survey statement 3: “There are known and unknown safety risks associated with the use of T&CM products”											
Strongly disagree	*n* (%)	1 (2)	8 (1)	3 (1)	17 (3)	7 (2)	6 (3)	3 (4)	1 (1)	4 (2)	50 (2)
Disagree		2 (3)	47 (9)	12 (6)	41 (7)	21 (6)	3 (2)	5 (6)	3 (4)	9 (9)	143 (6)
Neither agree nor disagree		7 (11)	39 (7)	25 (12)	131 (21)	41 (11)	27 (14)	12 (15)	16 (23)	25 (14)	323 (14)
Agree		17 (28)	375 (69)	59 (29)	181 (29)	135 (36)	53 (27)	34 (41)	19 (27)	48 (26)	921 (39)
Strongly agree		34 (56)	71 (13)	104 (51)	257 (41)	175 (46)	107 (55)	28 (34)	32 (45)	96 (53)	904 (39)
Survey statement 4: “T&CM products should be considered within the scope of professional pharmacy practice”											
Strongly disagree		1 (2)	6 (1)	5 (2)	17 (3)	9 (2)	1 (1)	4 (5)	3 (4)	0 (0.x)	46 (2)
Disagree		4 (7)	8 (1)	14 (7)	24 (4)	9 (2)	1 (1)	5 (6)	4 (6)	8 (4)	77 (3)
Neither agree nor disagree		6 (10)	12 (2)	23 (11)	113 (18)	60 (16)	13 (7)	12 (15)	16 (23)	26 (14)	281 (12)
Agree		18 (30)	339 (63)	57 (28)	170 (27)	120 (32)	50 (26)	16 (20)	19 (27)	43 (24)	832 (36)
Strongly agree		32 (52)	175 (32)	104 (51)	303 (48)	181 (48)	131 (67)	45 (55)	29 (41)	105 (58)	1,105 (47)
Survey statement 5: “Types of T&CM products that should be integrated into the professional practice of pharmacy” (more than 1 option can be chosen)											
Products containing herbal or botanical ingredients	*n* (%)	53 (87)	482 (89)	192 (95)	562 (90)	317 (84)	173 (88)	73 (89)	56 (79)	157 (86)	2,065 (88)
Nutritional products containing vitamins/minerals and amino acids		47 (77)	477 (88)	147 (72)	569 (91)	335 (88)	156 (80)	72 (88)	52 (73)	139 (76)	1,994 (85)
Non-vitamin and mineral nutritional supplements		42 (69)	421 (78)	171 (84)	532 (85)	325 (86)	140 (71)	73 (89)	39 (55)	112 (62)	1,855 (79)
Probiotic and prebiotic formulations		41 (67)	396 (73)	182 (90)	561 (89)	302 (80)	134 (68)	76 (93)	41 (58)	108 (59)	1,841 (79)
Homeopathic products		39 (64)	0 (0.x)	61 (30)	389 (62)	116 (31)	92 (47)	54 (66)	14 (20)	131 (72)	896 (38)
No T&CM products should be included in the professional practice of pharmacy		1 (2)	0 (0)	1 (0.x)	3 (0.x)	3 (1)	0 (0)	0 (0)	0 (0)	0 (0)	8 (0)

### 3.3 Pharmacists’ responsibilities toward the safe and proper use of traditional and complementary medicine products

Overall, as shown in Table 3, most of the participants in this study agreed or strongly agreed with all 18 responsibilities posed to them regarding the safe and proper use of T&CM products (ranging from 77% to 92%). Pharmacists in this study mostly agreed on *Responsibility 7* [pharmacists should be able to inform consumers about the potential risks associated with the use of T&CM products (e.g., side effects and drug–herb interactions)] with 2,151 participants (or 92%) (ranging from 85% in Lebanon to 98% in Carbo Verde) opting “agree” or “strongly agree.”

**TABLE 3:** Level of agreement with pharmacists’ responsibilities regarding the safe and proper use of traditional and complementary medicine (T&CM) products.

Level of agreement		Participating countries									Total (*n* = 2,341)
		Carbo Verde (*n* = 61)	China (*n* = 540)	Croatia (*n* = 203)	Lebanon (*n* = 627)	Malaysia (*n* = 379)	Philippines (*n* = 196)	Portugal (*n* = 82)	Saudi Arabia (*n* = 71)	Thailand (*n* = 182)	
*Responsibility 1*. “pharmacists are in a position to prevent harm associated with the inappropriate use of T&CM products”											
Strongly disagree	*n* (%)	3 (5)	4 (1)	5 (2)	21 (3)	4 (1)	1 (1)	3 (4)	5 (7)	1 (1)	47 (2)
									
Disagree		5 (8)	17 (3)	7 (3)	39 (6)	3 (1)	0 (0)	8 (10)	4 (6)	1 (1)	84 (4)
									
Neither agree nor disagree		7 (11)	24 (4)	12 (6)	144 (23)	16 (4)	7 (4)	9 (11)	9 (13)	13 (7)	241 (10)
									
Agree		19 (31)	262 (49)	43 (21)	156 (25)	64 (17)	22 (11)	23 (28)	16 (23)	45 (25)	650 (28)
									
Strongly agree		27 (44)	233 (43)	136 (67)	267 (43)	292 (77)	166 (85)	39 (48)	37 (52)	122 (67)	1,319 (56)
*Responsibility 2*. “Pharmacists are in a position to provide evidence-based advice about T&CM products to optimize patient outcomes”											
Strongly disagree	*n* (%)	2 (3)	1 (0.x)	4 (2)	22 (4)	2 (1)	1 (1)	2 (2)	5 (7)	0 (0)	39 (2)
Disagree		5 (8)	10 (2)	8 (4)	43 (7)	7 (2)	2 (1)	4 (5)	2 (3)	3 (2)	84 (4)
Neither agree nor disagree		6 (10)	7 (1)	14 (7)	169 (27)	22 (6)	10 (5)	15 (18)	13 (18)	13 (7)	269 (11)
Agree		17 (28)	267 (49)	43 (21)	169 (27)	84 (22)	28 (14)	23 (28)	20 (28)	47 (26)	698 (30)
Strongly agree		31 (51)	255 (47)	134 (66)	224 (36)	264 (70)	155 (79)	38 (46)	31 (44)	119 (65)	1,251 (53)
*Responsibility 3*. “Pharmacists should respect consumer’s autonomy regarding their choice to use T&CM products”											
Strongly disagree	*n* (%)	1 (2)	2 (0.x)	15 (7)	32 (5)	8 (2)	3 (2)	4 (5)	2 (3)	3 (2)	70 (3)
Disagree		4 (7)	16 (3)	28 (14)	36 (6)	10 (3)	5 (3)	9 (11)	3 (4)	8 (4)	119 (5)
Neither agree nor disagree		7 (11)	20 (4)	51 (25)	113 (18)	68 (18)	16 (8)	12 (15)	15 (21)	40 (22)	342 (15)
Agree		21 (34)	275 (51)	55 (27)	174 (28)	118 (31)	49 (25)	22 (27)	19 (27)	49 (27)	782 (33)
Strongly agree		28 (46)	227 (42)	54 (27)	272 (43)	175 (46)	123 (63)	35 (43)	32 (45)	82 (45)	1,028 (44)
*Responsibility 4*. “Pharmacists should routinely ask about the use of T&CM products by those taking pharmaceutical medicines”											
Strongly disagree	*n* (%)	1 (2)	3 (1)	5 (2)	11 (2)	5 (1)	1 (1)	2 (2)	2 (3)	0 (0)	30 (1)
Disagree		0 (0)	17 (3)	6 (3)	15 (2)	2 (1)	4 (2)	3 (4)	1 (1)	2 (1)	50 (2)
Neither agree nor disagree		2 (3)	20 (4)	19 (9)	96 (15)	16 (4)	14 (7)	9 (11)	6 (8)	13 (7)	195 (8)
Agree		11 (18)	304 (56)	55 (27)	136 (22)	76 (20)	45 (23)	18 (22)	15 (21)	50 (27)	710 (30)
Strongly agree		47 (77)	196 (36)	118 (58)	369 (59)	280 (74)	132 (67)	50 (61)	47 (66)	117 (64)	1,356 (58)
*Responsibility 5*. “Pharmacists should routinely document the use of T&CM products on patients’ records”											
Strongly disagree	*n* (%)	0 (0)	5 (1)	9 (4)	15 (2)	5 (1)	1 (1)	0 (0)	2 (3)	6 (3)	43 (2)
Disagree		0 (0)	23 (4)	10 (5)	37 (6)	7 (2)	2 (1)	2 (2)	4 (6)	7 (4)	92 (4)
Neither agree nor disagree		4 (7)	42 (8)	56 (28)	103 (16)	33 (9)	21 (11)	10 (12)	7 (10)	28 (15)	304 (13)
Agree		17 (28)	306 (57)	52 (26)	151 (24)	97 (26)	44 (22)	28 (34)	14 (20)	50 (27)	759 (32)
Strongly agree		40 (66)	164 (30)	76 (37)	321 (51)	237 (63)	128 (65)	42 (51)	44 (62)	91 (50)	1,143 (49)
*Responsibility 6*. “Pharmacists should be able to source and provide consumer medicine information (CMI) about T&CM products”											
Strongly disagree	*n* (%)	0 (0)	3 (1)	4 (2)	9 (1)	5 (1)	1 (1)	1 (1)	1 (1)	0 (0)	24 (1)
Disagree		0 (0)	41 (8)	3 (1)	22 (4)	2 (1)	4 (2)	1 (1)	4 (6)	0 (0)	77 (3)
Neither agree nor disagree		1 (2)	46 (9)	9 (4)	86 (14)	43 (11)	10 (5)	6 (7)	12 (17)	14 (8)	227 (10)
Agree		12 (20)	305 (56)	32 (16)	170 (27)	112 (30)	37 (19)	12 (15)	17 (24)	40 (22)	737 (31)
Strongly agree		48 (79)	145 (27)	155 (76)	340 (54)	217 (57)	144 (73)	62 (76)	37 (52)	128 (70)	1,276 (55)
*Responsibility 7*. “Pharmacists should be able to inform consumers about the potential risks associated with the use of T&CM products (e.g., side effects, drug–herb interactions)”											
Strongly disagree	*n* (%)	0 (0)	1 (0)	3 (1)	13 (2)	2 (1)	1 (1)	2 (2)	0 (0)	0 (0)	22 (1)
Disagree		1 (2)	13 (2)	3 (1)	10 (2)	4 (1)	0 (0)	1 (1)	2 (3)	0 (0)	34 (1)
Neither agree nor disagree		0 (0)	18 (3)	5 (2)	69 (11)	13 (3)	6 (3)	3 (4)	8 (11)	12 (7)	134 (6)
Agree		5 (8)	286 (53)	14 (7)	107 (17)	87 (23)	22 (11)	10 (12)	16 (23)	28 (15)	575 (25)
Strongly agree		55 (90)	222 (41)	178 (88)	428 (68)	273 (72)	167 (85)	66 (80)	45 (63)	142 (78)	1,576 (67)
*Responsibility 8*. “Pharmacists must be able to provide advice based on accurate information (e.g., scientific studies) on the effectiveness of T&CM products”											
Strongly disagree	*n* (%)	0 (0)	1 (0.x)	2 (1)	9 (1)	5 (1)	2 (1)	1 (1)	0 (0)	0 (0)	20 (1)
Disagree		0 (0)	12 (2)	4 (2)	20 (3)	3 (1)	0 (0)	2 (2)	1 (1)	1 (1)	43 (2)
Neither agree nor disagree		3 (5)	17 (3)	15 (7)	78 (12)	27 (7)	3 (2)	3 (4)	12 (17)	15 (8)	173 (7)
Agree		10 (16)	312 (58)	26 (13)	121 (19)	99 (26)	36 (18)	9 (11)	14 (20)	32 (18)	659 (28)
Strongly agree		48 (79)	198 (37)	156 (77)	399 (64)	245 (65)	155 (79)	67 (82)	44 (62)	134 (74)	1,446 (62)
*Responsibility 9*. “Pharmacists should be able to provide their patients personalized advice about the use of T&CM products”											
Strongly disagree	*n* (%)	0 (0)	1 (0)	3 (1)	16 (3)	2 (1)	1 (1)	2 (2)	1 (1)	0 (0)	26 (1)
Disagree		0 (0)	8 (1)	3 (1)	20 (3)	8 (2)	4 (2)	1 (1)	3 (4)	0 (0)	47 (2)
Neither agree nor disagree		3 (5)	12 (2)	13 (6)	76 (12)	42 (11)	14 (7)	2 (2)	11 (15)	11 (6)	184 (8)
Agree		11 (18)	315 (58)	34 (17)	140 (22)	102 (27)	38 (19)	15 (18)	19 (27)	36 (20)	710 (30)
Strongly agree		47 (77)	204 (38)	150 (74)	375 (60)	225 (59)	139 (71)	62 (76)	37 (52)	135 (74)	1,374 (59)
*Responsibility 10*. “Pharmacists should be able to guide consumers (including patients) in making an informed decision about the use of T&CM products”											
Strongly disagree	*n* (%)	0 (0)	1 (0.x)	4 (2)	13 (2)	3 (1)	1 (1)	2 (2)	3 (4)	0 (0)	27 (1)
Disagree		1 (2)	7 (1)	3 (1)	14 (2)	5 (1)	0 (0)	1 (1)	4 (6)	0 (0)	35 (1)
Neither agree nor disagree		1 (2)	15 (3)	8 (4)	94 (15)	27 (7)	6 (3)	4 (5)	8 (11)	11 (6)	174 (7)
Agree		10 (16)	311 (58)	32 (16)	154 (25)	105 (28)	36 (18)	12 (15)	19 (27)	35 (19)	714 (30)
Strongly agree		49 (80)	206 (38)	156 (77)	352 (56)	239 (63)	153 (78)	63 (77)	37 (52)	136 (75)	1,391 (59)
*Responsibility 11*. “Pharmacists should monitor for any the adverse events associated with the use of T&CM products”											
Strongly disagree	*n* (%)	0 (0)	3 (1)	3 (1)	9 (1)	7 (2)	1 (1)	0 (0)	2 (3)	5 (3)	30 (1)
Disagree		0 (0)	18 (3)	3 (1)	21 (3)	7 (2)	5 (3)	2 (2)	1 (1)	2 (1)	59 (3)
Neither agree nor disagree		1 (2)	30 (6)	18 (9)	88 (14)	45 (12)	13 (7)	5 (6)	13 (18)	20 (11)	233 (10)
Agree		14 (23)	302 (56)	48 (24)	158 (25)	107 (28)	26 (13)	16 (20)	19 (27)	43 (24)	733 (31)
Strongly agree		46 (75)	187 (35)	131 (65)	351 (56)	213 (56)	151 (77)	59 (72)	36 (51)	112 (62)	1286 (55)
*Responsibility 12*. “Pharmacists should be able to provide guidance on the management of adverse events associated with the use of T&CM products when they occur”											
Strongly disagree	*n* (%)	0 (0)	4 (1)	4 (2)	10 (2)	4 (1)	2 (1)	1 (1)	2 (3)	0 (0)	27 (1)
Disagree		0 (0)	5 (1)	5 (2)	18 (3)	8 (2)	0 (0)	2 (2)	0 (0)	0 (0)	38 (2)
Neither agree nor disagree		0 (0)	21 (4)	8 (4)	75 (12)	36 (9)	9 (5)	4 (5)	11 (15)	14 (8)	178 (8)
Agree		12 (20)	317 (59)	39 (19)	173 (28)	105 (28)	26 (13)	9 (11)	22 (31)	39 (21)	742 (32)
Strongly agree		49 (80)	193 (36)	147 (72)	351 (5)	226 (60)	159 (81)	66 (80)	36 (51)	129 (71)	1,356 (58)
*Responsibility 13*. “Pharmacists should report suspected adverse events associated with the use of T&CM products to the relevant medicines’ regulatory authority”											
Strongly disagree	*n* (%)	0 (0)	2 (0.x)	4 (2)	15 (2)	4 (1)	2 (1)	0 (0)	3 (4)	3 (2)	33 (1)
Disagree		0 (0)	6 (1)	3 (1)	21 (3)	5 (1)	0 (0)	1 (1)	1 (1)	2 (1)	39 (2)
Neither agree nor disagree		0 (0)	21 (4)	8 (4)	84 (13)	13 (3)	5 (3)	3 (4)	10 (14)	20 (11)	164 (7)
Agree		7 (11)	290 (54)	30 (15)	157 (25)	78 (21)	27 (14)	10 (12)	14 (20)	41 (23)	654 (28)
Strongly agree		54 (89)	221 (41)	158 (78)	350 (56)	279 (74)	162 (83)	68 (83)	43 (61)	116 (64)	1,451 (62)
*Responsibility 14*. “Pharmacists should refer consumers (including patients) to qualified T&CM practitioners for advice about T&CM product use”											
Strongly disagree	*n* (%)	2 (3)	1 (0.x)	17 (8)	14 (2)	9 (2)	4 (2)	8 (10)	2 (3)	10 (5)	67 (3)
Disagree		2 (3)	7 (1)	20 (10)	31 (5)	12 (3)	2 (1)	11 (13)	2 (2)	14 (8)	101 (4)
Neither agree nor disagree		3 (5)	6 (1)	51 (25)	148 (24)	46 (12)	14 (7)	22 (27)	21 (30)	36 (20)	347 (15)
Agree		13 (21)	281 (52)	52 (26)	161 (26)	102 (27)	39 (20)	20 (24)	16 (23)	44 (24)	728 (31)
Strongly agree		41 (67)	245 (45)	63 (31)	273 (44)	210 (55)	137 (70)	21 (26)	30 (42)	78 (43)	1,098 (47)
*Responsibility 15*. “Pharmacists should only stock T&CM products approved by the local regulatory authority such as the drug regulatory authorities”											
Strongly disagree	*n* (%)	3 (5)	4 (1)	9 (4)	30 (5)	6 (2)	1 (1)	6 (7)	2 (3)	6 (3)	67 (3)
Disagree		1 (2)	39 (7)	14 (7)	34 (5)	9 (2)	1 (1)	5 (6)	1 (1)	2 (1)	106 (5)
Neither agree nor disagree		1 (2)	51 (9)	19 (9)	85 (14)	19 (5)	8 (4)	5 (6)	7 (10)	17 (9)	212 (9)
Agree		7 (11)	253 (47)	42 (21)	122 (19)	51 (13)	22 (11)	14 (17)	17 (24)	19 (10)	547 (23)
Strongly agree		49 (80)	193 (36)	119 (59)	356 (57)	294 (78)	164 (84)	52 (63)	44 (62)	138 (76)	1,409 (60)
*Responsibility 16*. “Business owners should set up the pharmacy so advice from a pharmacist can be easily accessed by those purchasing T&CM products”											
Strongly disagree	*n* (%)	0 (0)	3 (1)	4 (2)	19 (3)	9 (2)	3 (2)	3 (4)	1 (1)	5 (3)	47 (2)
Disagree		1 (2)	8 (1)	9 (4)	20 (3)	10 (3)	3 (2)	2 (2)	6 (8)	4 (2)	63 (3)
Neither agree nor disagree		0 (0)	7 (1)	39 (19)	101 (16)	47 (12)	15 (8)	10 (12)	12 (17)	29 (16)	260 (11)
Agree		10 (16)	270 (50)	56 (28)	178 (28)	91 (24)	40 (20)	25 (30)	14 (20)	44 (24)	728 (31)
Strongly agree		50 (82)	252 (47)	95 (47)	309 (49)	222 (59)	135 (69)	42 (51)	38 (54)	100 (55)	1,243 (53)
*Responsibility 17*. “Pharmacists should provide training for their staff about T&CM products”											
Strongly disagree	*n* (%)	0 (0)	2 (0)	2 (1)	15 (2)	5 (1)	1 (1)	0 (0)	2 (3)	3 (2)	30 (1)
Disagree		0 (0)	10 (2)	7 (3)	22 (4)	1 (0)	1 (1)	1 (1)	2 (3)	1 (1)	45 (2)
Neither agree nor disagree		1 (2)	11 (2)	11 (5)	83 (13)	16 (4)	9 (5)	5 (6)	12 (17)	23 (13)	171 (7)
Agree		9 (15)	293 (54)	31 (15)	167 (27)	98 (26)	39 (20)	14 (17)	18 (25)	40 (22)	709 (30)
Strongly agree		51 (84)	224 (41)	152 (75)	340 (54)	259 (68)	146 (74)	62 (76)	37 (52)	115 (63)	1,386 (59)
*Responsibility 18*. “Pharmacists should communicate with other healthcare professionals regarding a patient’s use of T&CM products to ensure appropriate and safe use”											
Strongly disagree	*n* (%)	0 (0)	3 (1)	4 (2)	13 (2)	3 (1)	1 (1)	0 (0)	2 (3)	1 (1)	27 (1)
Disagree		0 (0)	8 (1)	6 (3)	11 (2)	2 (1)	1 (1)	1 (1)	2 (3)	1 (1)	32 (1)
Neither agree nor disagree		1 (2)	5 (1)	20 (10)	96 (15)	18 (5)	9 (5)	6 (7)	10 (14)	14 (8)	179 (8)
Agree		9 (15)	282 (52)	51 (25)	169 (27)	82 (22)	25 (13)	20 (24)	17 (24)	38 (21)	693 (30)
Strongly agree		51 (84)	242 (45)	122 (60)	338 (54)	274 (72)	160 (82)	55 (67)	40 (56)	128 (70)	1,410 (60)

This was followed by *Responsibility 8* [pharmacists must be able to provide advice based on accurate information (e.g., scientific studies) on the effectiveness of T&CM products]; *Responsibility 10* (pharmacists should be able to guide consumers (including patients) in making an informed decision about the use of T&CM products); *Responsibility 12* (pharmacists should be able to provide guidance on the management of adverse events associated with the use of T&CM products when they occur); *Responsibility 13* (pharmacists should report suspected adverse events associated with the use of T&CM products to the relevant medicines regulatory authority); and *Responsibility 18* (pharmacists should communicate with other healthcare professionals regarding a patient’s use of T&CM products to ensure appropriate and safe use), on which 90% of the participants agreed separately (ranging 82%–97%, 79%–97%, 82%–100%, 80%–100%, and 80%–98% for *Responsibilities 8, 10, 12, 13,* and *18*, respectively).

Other responsibilities, such as *Responsibility 9* (pharmacists should be able to provide their patients personalized advice about the use of T&CM products) and *Responsibility 17* (pharmacists should provide training for their staff about T&CM products), were also greatly agreed on by 89% of the participants separately (ranging 79%–96% and 77%–98% for *Responsibilities 9* and *17*, respectively).

Responsibilities least agreed on were *Responsibility 3* (pharmacists should respect consumer’s autonomy regarding their choice to use T&CM products), with 1,810 participants (or 77%) (ranging from 54% in Croatia to 93% in China) agreeing on it, and *Responsibility 14* (pharmacists should refer consumers (including patients) to qualified T&CM practitioners for advice about T&CM product use), with 1,826 participants (or 78%) (ranging from 50% in Portugal and 97% in China) agreeing on it. The overall and country results regarding the level of agreement with all the suggested responsibilities toward the safe and proper use of T&CM products are shown in Table 3.

### 3.4 Pharmacists’ views about the action and support required for pharmacists to develop a professional role related to T&CM products

Overall, as shown in Table 4, most of the participants in this study agreed or strongly agreed with all the 13 actions proposed to support pharmacists in developing a professional role related to T&CM products (ranging from 67% to 91%). Pharmacist participants in this study mostly agreed on *Action 5* (there is a need to provide T&CM product education as continuing professional development) and *Action 11* (improvements in the regulatory standards of T&CM product quality, safety and efficacy are required), on which 91% of the participants agreed separately (ranging from 82% in Lebanon to 98% in Thailand and from 82% in Lebanon to 98% in China for *Actions 5* and *11*, respectively).

**TABLE 4:** Level of agreement with actions and support required for pharmacists to develop a professional role related to traditional and complementary medicine (T&CM) products.

Level of agreement		Participating countries									Total (*n* = 2,341)
		Carbo Verde (*n* = 61)	China (*n* = 540)	Croatia (*n* = 203)	Lebanon (*n* = 627)	Malaysia (*n* = 379)	Philippines (*n* = 196)	Portugal (*n* = 82)	Saudi Arabia (*n* = 71)	Thailand (*n* = 182)	
*Action 1*: “There is a need to clearly define pharmacists’ professional responsibilities associated with T&CM products”											
Strongly disagree	*n* (%)	0 (0)	8 (1)	3 (1)	12 (2)	5 (1)	2 (1)	1 (1)	1 (1)	1 (1)	33 (1)
Disagree		0 (0)	25 (5)	3 (1)	19 (3)	8 (2)	2 (1)	1 (1)	4 (6)	0 (0)	62 (3)
Neither agree nor disagree		1 (2)	22 (4)	20 (10)	75 (12)	26 (7)	6 (3)	13 (16)	10 (14)	17 (9)	190 (8)
Agree		13 (21)	313 (58)	35 (17)	164 (26)	87 (23)	27 (14)	20 (24)	14 (20)	45 (25)	718 (31)
Strongly agree		47 (77)	172 (32)	142 (70)	357 (57)	253 (67)	159 (81)	47 (57)	42 (59)	119 (65)	1,338 (57)
*Action 2*: “There is a need to develop T&CM product practice standards for pharmacists”											
Strongly disagree	*n* (%)	0 (0)	2 (0)	3 (1)	12 (2)	4 (1)	1 (1)	1 (1)	1 (1)	0 (0)	24 (1)
Disagree		2 (3)	14 (3)	4 (2)	15 (2)	6 (2)	2 (1)	3 (4)	0 (0)	1 (1)	47 (2)
Neither agree nor disagree		2 (3)	20 (4)	12 (6)	87 (14)	26 (7)	10 (5)	5 (6)	11 (15)	18 (10)	191 (8)
Agree		7 (11)	322 (60)	46 (23)	178 (28)	96 (25)	34 (17)	16 (20)	20 (28)	43 (24)	762 (33)
Strongly agree		50 (82)	182 (34)	138 (68)	335 (53)	247 (65)	149 (76)	57 (70)	39 (55)	120 (66)	1,317 (56)
*Action 3*: “There is a need to develop an accreditation system for pharmacists’ T&CM competency”											
Strongly disagree	*n* (%)	1 (2)	4 (1)	5 (2)	17 (3)	4 (1)	4 (2)	3 (4)	0 (0)	0 (0)	38 (2)
Disagree		1 (2)	19 (4)	6 (3)	19 (3)	9 (2)	5 (3)	3 (4)	5 (7)	2 (1)	69 (3)
Neither agree nor disagree		2 (3)	30 (6)	31 (15)	118 (19)	34 (9)	17 (9)	12 (15)	11 (15)	25 (14)	280 (12)
Agree		12 (20)	308 (57)	59 (29)	186 (30)	94 (25)	44 (22)	22 (27)	18 (25)	39 (21)	782 (33)
Strongly agree		45 (74)	179 (33)	102 (50)	287 (46)	238 (63)	126 (64)	42 (51)	37 (52)	116 (64)	1,172 (50)
*Action 4*: “There is a need to include T&CM product education in undergraduate professional pharmacy curricula”											
Strongly disagree	*n* (%)	0 (0)	3 (1)	3 (1)	20 (3)	3 (1)	2 (1)	2 (2)	2 (3)	0 (0)	35 (1)
Disagree		1 (2)	9 (2)	5 (2)	21 (3)	3 (1)	2 (1)	2 (2)	0 (0)	0 (0)	43 (2)
Neither agree nor disagree		3 (5)	13 (2)	12 (6)	72 (11)	23 (6)	10 (5)	8 (10)	12 (17)	5 (3)	158 (7)
Agree		8 (13)	310 (57)	38 (19)	166 (26)	90 (24)	37 (19)	16 (20)	19 (27)	35 (19)	719 (31)
Strongly agree		49 (80)	205 (38)	145 (71)	348 (56)	260 (69)	145 (74)	54 (66)	38 (34)	142 (78)	1,386 (59)
*Action 5*: “There is a need to provide T&CM product education as continuing professional development”											
Strongly disagree	*n* (%)	1 (2)	3 (1)	2 (1)	10 (2)	3 (1)	1 (1)	2 (2)	1 (1)	0 (0)	23 (1)
Disagree		0 (0)	8 (1)	3 (1)	21 (3)	2 (1)	1 (1)	2 (2)	2 (3)	0 (0)	39 (2)
Neither agree nor disagree		2 (3)	8 (1)	10 (5)	84 (13)	22 (6)	12 (6)	3 (4)	7 (10)	3 (2)	151 (6)
Agree		6 (10)	285 (53)	31 (15)	187 (30)	85 (22)	38 (19)	14 (17)	19 (27)	31 (17)	696 (30)
Strongly agree		52 (85)	236 (44)	157 (77)	325 (52)	267 (70)	144 (73)	61 (74)	42 (59)	148 (81)	1,432 (61)
*Action 6*: “In the workplace, there is a need to improve access to evidence-based T&CM information”											
Strongly disagree	*n* (%)	0 (0)	1 (0)	2 (1)	13 (2)	4 (1)	1 (1)	1 (1)	2 (3)	0 (0)	24 (1)
Disagree		0 (0)	8 (1)	4 (2)	23 (4)	2 (1)	0 (0)	3 (4)	2 (3)	0 (0)	42 (2)
Neither agree nor disagree		2 (3)	13 (2)	14 (7)	94 (15)	18 (5)	10 (5)	4 (5)	8 (11)	13 (7)	176 (8)
Agree		11 (18)	317 (59)	30 (15)	174 (28)	88 (23)	43 (22)	16 (20)	15 (21)	33 (18)	727 (31)
Strongly agree		48 (79)	201 (37)	153 (75)	323 (52)	267 (70)	142 (72)	58 (71)	44 (62)	136 (75)	1,372 (59)
*Action 7*: “In the workplace, there is a need to provide support for pharmacists to undertake training in T&CM”											
Strongly disagree	*n* (%)	1 (2)	2 (0)	4 (2)	10 (2)	3 (1)	1 (1)	1 (1)	1 (1)	0 (0)	23 (1)
Disagree		1 (2)	8 (1)	4 (2)	21 (3)	5 (1)	4 (2)	2 (2)	2 (3)	0 (0)	47 (2)
Neither agree nor disagree		1 (2)	8 (1)	22 (11)	94 (15)	27 (7)	11 (6)	10 (12)	10 (14)	11 (6)	194 (8)
Agree		9 (15)	283 (52)	38 (19)	183 (29)	96 (25)	41 (21)	19 (23)	18 (25)	31 (17)	718 (31)
Strongly agree		49 (80)	239 (44)	135 (67)	319 (51)	248 (65)	139 (71)	50 (61)	40 (56)	140 (77)	1,359 (58)
*Action 8*: “In the workplace, pharmacy business owners should employ accredited practitioners (e.g., naturopath - herbalist - nutritionist) to provide advice to consumers about T&CM products”											
Strongly disagree	*n* (%)	8 (13)	1 (0)	71 (35)	52 (8)	14 (4)	14 (7)	23 (28)	5 (7)	1 (1)	189 (8)
Disagree		9 (15)	12 (2)	42 (21)	65 (10)	14 (4)	17 (9)	14 (17)	4 (6)	2 (1)	179 (8)
Neither agree nor disagree		14 (23)	21 (4)	47 (23)	154 (25)	82 (22)	38 (19)	20 (24)	13 (18)	23 (13)	412 (18)
Agree		17 (28)	294 (54)	24 (12)	146 (23)	98 (26)	51 (26)	17 (21)	18 (25)	37 (20)	702 (30)
Strongly agree		13 (21)	212 (39)	19 (9)	210 (33)	171 (45)	76 (39)	8 (10)	31 (44)	119 (65)	859 (37)
*Action 9*: “More support is required to facilitate non-biased research about the efficacy and safety of T&CM products”											
Strongly disagree	*n* (%)	0 (0)	1 (0.x)	2 (1)	9 (1)	1 (0.x)	2 (1)	0 (0)	3 (4)	0 (0)	18 (1)
Disagree		0 (0)	4 (1)	6 (3)	20 (3)	4 (1)	1 (1)	6 (7)	2 (3)	0 (0)	43 (2)
Neither agree nor disagree		2 (3)	7 (1)	23 (11)	115 (18)	15 (4)	16 (8)	11 (13)	9 (13)	10 (5)	208 (9)
Agree		8 (13)	304 (56)	60 (30)	178 (28)	84 (22)	43 (22)	15 (18)	17 (24)	24 (13)	733 (31)
Strongly agree		51 (84)	224 (41)	112 (55)	305 (49)	275 (73)	134 (68)	50 (61)	40 (56)	148 (81)	1,339 (57)
*Action 10*: “There is a need to develop effective interprofessional communication processes regarding T&CM product use”											
Strongly disagree	*n* (%)	0 (0)	1 (0.x)	3 (1)	10 (2)	2 (1)	1 (1)	1 (1)	2 (3)	1 (1)	21 (1)
Disagree		1 (2)	10 (2)	6 (3)	13 (2)	3 (1)	2 (1)	2 (2)	2 (3)	0 (0)	39 (2)
Neither agree nor disagree		2 (3)	14 (3)	34 (17)	121 (19)	14 (4)	9 (5)	8 (10)	8 (11)	9 (5)	219 (9)
Agree		12 (20)	319 (59)	53 (26)	186 (30)	105 (28)	43 (22)	19 (23)	22 (31)	38 (21)	797 (34)
Strongly agree		46 (75)	196 (36)	107 (53)	297 (47)	255 (67)	141 (72)	52 (63)	37 (52)	134 (74)	1,265 (54)
*Action 11*: “Improvement in the regulatory standards of T&CM product quality, safety and efficacy are required”											
Strongly disagree	*n* (%)	0 (0)	1 (0)	2 (1)	7 (1)	2 (1)	1 (1)	0 (0)	0 (0)	0 (0)	13 (1)
Disagree		0 (0)	6 (1)	4 (2)	21 (3)	2 (1)	1 (1)	1 (1)	1 (1)	0 (0)	36 (2)
Neither agree nor disagree		3 (5)	6 (1)	21 (10)	84 (13)	14 (4)	8 (4)	9 (11)	7 (10)	16 (9)	168 (7)
Agree		13 (21)	285 (53)	42 (21)	184 (29)	87 (23)	34 (17)	7 (9)	15 (21)	33 (18)	700 (30)
Strongly agree		45 (74)	242 (45)	134 (66)	331 (53)	274 (72)	152 (78)	65 (79)	48 (68)	133 (73)	1,424 (61)
*Action 12*: “Improvements in the regulatory standards of T&CM product labelling and advertising are required”											
Strongly disagree	*n* (%)	0 (0)	1 (0.x)	3 (1)	5 (1)	2 (1)	1 (1)	0 (0)	1 (1)	0 (0)	13 (1)
Disagree		0 (0)	7 (1)	2 (1)	22 (4)	2 (1)	0 (0)	2 (2)	3 (4)	0 (0)	38 (2)
Neither agree nor disagree		3 (5)	7 (1)	22 (11)	103 (16)	18 (5)	6 (3)	7 (9)	10 (14)	17 (9)	193 (8)
Agree		16 (26)	282 (52)	45 (22)	184 (29)	77 (20)	32 (16)	11 (13)	14 (20)	27 (15)	688 (29)
Strongly agree		42 (69)	243 (45)	131 (65)	313 (50)	280 (74)	157 (80)	62 (76)	43 (61)	138 (76)	1,409 (60)
*Action 13*: “Regulatory bodies should consider scheduling of T&CM products as ‘pharmacy only’ medicines”											
Strongly disagree	*n* (%)	1 (2)	3 (1)	4 (2)	16 (3)	11 (3)	3 (2)	2 (2)	3 (4)	4 (2)	47 (2)
Disagree		4 (7)	19 (4)	8 (4)	31 (5)	26 (7)	3 (2)	8 (10)	6 (8)	9 (5)	114 (5)
Neither agree nor disagree		2 (3)	43 (8)	18 (9)	127 (20)	79 (21)	19 (10)	8 (10)	17 (24)	29 (16)	342 (15)
Agree		18 (30)	265 (49)	39 (19)	149 (24)	94 (25)	49 (25)	20 (24)	15 (21)	34 (19)	683 (29)
Strongly agree		36 (59)	210 (39)	134 (66)	304 (48)	169 (45)	122 (62)	44 (54)	30 (42)	106 (58)	1,155 (49)

This was followed by *Action 4* (there is a need to include T&CM product education in undergraduate professional pharmacy curricula), *Action 6* (in the workplace, there is a need to improve access to evidence-based T&CM information), and *Action 12* (improvements in the regulatory standards of T&CM product labelling and advertising are required), on which 90% of the participants agreed separately (ranging from 80% in Saudi Arabia to 97% in Thailand, 79% in Lebanon to 97% in Carbo Verde, and 79% in Lebanon to 97% in China for *Actions 4, 6,* and *12*, respectively).

Other actions, such as *Action 2* (there is a need to develop T&CM product practice standards for pharmacists), *Action 7* (in the workplace, there is a need to provide support for pharmacists to undertake training in T&CM), and *Action 9* (more support is required to facilitate non-biased research about the efficacy and safety of T&CM products) were also agreed on by 89% of the participants separately (ranging 82%–93%, 80%–95%, and 77%–98% for *Actions 2, 7,* and *9*, respectively).

Other actions considered to be highly important were *Action 1* (there is a need to clearly define pharmacists’ professional responsibilities associated with T&CM products) and *Action 10* (there is a need to develop effective interprofessional communication processes regarding T&CM product use) on which 88% of the participants agreed separately.

Actions which were least agreed on were *Action 8* (in the workplace, pharmacy business owners should employ accredited practitioners (e.g., naturopath—herbalist—nutritionist) to provide advice to consumers about T&CM products), with 1,561 participants (or 67%) (ranging from 21% in Croatia to 94% in China) agreeing on it, and *Action 13* (regulatory bodies should consider scheduling of T&CM products as “pharmacy only” medicines), with 1,838 participants (or 79%) (ranging from 63% in Saudi Arabia to 89% in Carbo Verde) agreeing on it.

## 4 Discussion

In this study, a set of professional practice responsibilities and actions that would enable pharmacists’ contribution to ensuring the quality and safe use of T&CM products were proposed to pharmacists across nine countries. This is the first study to examine the pharmacist’s role in T&CM products across a range of countries during a single 12-month period (2022). The data obtained from 2,341 pharmacists provide a contemporary, multi-country representation and indicate an overall agreement on broadening the scope of professional practice to assume responsibilities associated with T&CM products. Together with previous research conducted over the last 5 years in Australia (Ung et al., 2018; Popattia and La Caze, 2021), China (Yao et al., 2020), and the United States (Harnett et al., 2019) that examined pharmacists’ views about their role and T&CM products, there are strong signals that formalizing and supporting pharmacists to enact such responsibilities in the real-world setting are palpable and needed. Therefore, concerned stakeholders, especially public health authorities and educational bodies, are expected to develop effective interventions to promote the professional role of pharmacists in ensuring the safe and proper use of T&CM products (Hijazi et al., 2021).

Consistent agreement across countries about the need for education and training, development of competency standards, establishment of accreditation of pharmacists’ services, and greater regulation of T&CM products and services provided by pharmacists should be considered a priority. Successful development of education, accreditation standards, and advancing quality assurance and regulatory frameworks would have positive implications for ensuring the appropriate and safe use of T&CM products by the public. The main advantage of a cross-country joint effort is that the attention to pharmacists’ role in T&CM products is universal, and the study findings allow some common ground for further discussion about pharmacists’ practice at an international level, which is a key determinant of making progress in a collaborative manner. Other advantages are that the study findings may draw further attention from other countries, their engagement in this topic, and international harmonization of the actions recommended. The responsibilities outlined here can contribute to establishing a common goal and developing a set of well-orchestrated actions that need to be undertaken by various stakeholders, whether pharmacists themselves, professional organizations, universities, government, consumer groups, or regulatory bodies (Ung et al., 2018).

Discussions about broadening pharmacists’ professional role in ensuring the safe and appropriate use of T&CM products have been described in detail twice over the last decade, and strategies required to accomplish this goal have been proposed (Ung et al., 2017b). A strategic model was proposed by Ung et al. (2018), and in 2020, an implementation science framework was recommended as an approach to navigating the multiple stakeholders and factors that influence pharmacists in successfully adopting responsibilities (Harnett et al., 2020). Despite the strategies proposed, including developments in education, day-to-day practice behaviors did not reflect that pharmacists are educated or proficient in inquiring or responding to T&CM use (Lee et al., 2021). The many challenges reported in the literature reflect that pharmacists remain under-resourced through a lack of education and training, access to reliable, reputable information, being time-poor, and concerns about the quality of T&CM products (Ung et al., 2017b). Such barriers often lead to inappropriate awareness of and recommendations for the use of T&CM products (Jalil et al., 2022), and, should they remain, will further stall the necessary developments in the provision of quality advice and products that support appropriate and safe use of T&CM products (Ung et al., 2017a).

An important consideration moving forward is that although 83% of the pharmacists surveyed in this study agreed that T&CM product use should be included in their scope of professional practice, the types of T&CM products for inclusion in their practice varied between countries. This is also not surprising considering the way different T&CM products are incorporated into health systems, as reflected in Supplementary File S1 by the nine studies included in this study. They may be part of traditional health-seeking behavior, such as in China (Li et al., 2017) and Cabo Verde (de Pina Araujo et al., 2022), whereby traditional medicine is highly integrated into the overall healthcare system, or as a complementary approach as seen in Portugal (Tavares, 2015) and Saudi Arabia (Khalil et al., 2018). Interestingly, of the five categories of T&CM products, there was a high level of agreement that herbal products should be included in pharmacists’ scope of professional practice regardless of the country and the level of integration into the healthcare system. However, there were substantial variations in agreement on the inclusion of T&CM products containing vitamins/minerals, non-vitamin/mineral nutrition ingredients, and pro- and pre-biotics into professional practice. This disparity may relate to these products sitting within the discipline of nutrition and dietetics rather than pharmaceuticals. Recent reports have indicated that pharmacists in Western countries are also keen on knowledge about nutrition (diet- and or nutrition-related supplements) (Carter et al., 2022a; Carter et al., 2022b).

Furthermore, although Australia, New Zealand, and the United States did not participate in this survey, it is interesting to note that previous research has reported that vitamin, mineral, and nutritional supplements account for the majority of T&CM products used in those countries (Harnett et al., 2023). The inclusion of homeopathic products was least supported, and the opinions were mostly diverse, which partly reflected the pharmacists’ acceptability and confidence in such products considering the current availability (or lack of) of clear evidence of homeopathy efficacy over placebo. These inherent nuances between the East and West and the types of T&CM used within the respective cultural settings should be considered when developing an international consensus. The need to reflect the diverse needs of the communities that pharmacists serve while maintaining an evidence-based approach to providing information and care is significant.

Providing evidence-based information about the safe use of medicine is central to good patient-centered care (Sackett et al., 1996). The Hippocratic Oath of “above all do no harm” shared among health professionals revealed itself strongly in relation to pharmacists’ level of agreement on T&CM responsibilities associated with safety. This is unsurprising, given that one of the central roles of the pharmacist is the custodian of drug safety. In particular, informing consumers about the potential risks associated with the use of T&CM products (*Responsibility 7*), providing advice about the effectiveness of T&CM products based on scientific sources, helping consumers in making informed decisions (*Responsibilities 8, 9, and 10*), providing guidance on managing adverse events associated with T&CM products (*Responsibility 12*), and reporting any suspected adverse events to the regulatory authority (*Responsibility 13*) received high levels of agreement. Pharmacists in this study indicated a willingness to work more closely and train other pharmacy staff (*Responsibility 17*) and communicate with other healthcare professionals (*Responsibility 18*). By taking a more holistic and patient-centered approach, pharmacists can, as a leading role in the multi-disciplinary healthcare team, help drive an improvement in the safe and appropriate use of T&CM products, which is an area often overlooked by others (Nguyen et al., 2019).

From a pragmatic point of view, these are well-established responsibilities practiced by pharmacists all over the world every day in relation to pharmaceutical drugs. Extending these responsibilities to T&CM products would appear at face value to be sensible. However, despite the encouraging levels of agreement with the responsibilities proposed to the pharmacists in this study, without adequate education and training in the relevant areas related to safety, as learned from other areas in advancing pharmacists’ practice, such recommendations of responsibilities become unrealistic and idealistic expectations (Forsyth and Rushworth, 2021). This concern is supported by the fact that nearly 70% of pharmacists surveyed in this study indicated that T&CM products were common in their community; however, only one-third found that undergraduate pharmacy education in T&CM was comprehensive enough to support their professional practice. This aligns with a strong agreement with the proposition of a need for training and education for both pharmacy students (*Action 4*) and continuing professional development (*Action 5*). The infamous words of Nelson Mandela, “*Education is the most powerful weapon to change the world*” (Mandela, 1990), are likely very relevant here. Very little can change in this area without the educational institutions involved in the training of pharmacists and the professional associations involved in governing professional development investing time and resources in the development of high-quality non-bias T&CM education. To this end, the current qualification system for pharmacists, as seen in the participating countries, offers multiple opportunities to introduce education and training interventions. As shown in Supplementary File S1, it is common that the minimum education requirements in the form of a university degree before registration as a pharmacist and the requirements for continuing education as a criterion for registration renewal are in place. Therefore, introducing and integrating T&CM-related education and training before and after registration should be considered a priority of actions to facilitate the improvement of pharmacists’ professional practice in the area of T&CM.

Adding another layer to the complexities for pharmacists adopting responsibilities and central to this argument is the need to access effective and safe T&CM products of known quality (Barnes, 2019). This was echoed by the pharmacists in this study, who supported the need for regulatory standards of T&CM products to better safeguard the quality, safety, and efficacy (*Action 11*) and how the labeling and advertising of T&CM products should be overseen more rigorously (*Action 12*). Another quality aspect is product-specific evidence, which is currently lacking (Barnes, 2019), with variations in the herbal constituents present between batches and brands yet using generic evidence for therapeutic claims (Cohen and Hunter, 2017). Not surprisingly, pharmacists are also seeking non-biased high-quality research to develop scientific evidence about the efficacy and safety of T&CM products (*Action 9*), which was also considered highly important by the broader scientific community (Gagnier et al., 2006). Confounding the need for greater regulation of T&CM products and services is the right of consumers to make decisions about their health and access the products and health services they prefer as part of broader cultural and personal beliefs and preferences (Ung et al., 2017b). Over-interference by regulators and healthcare professionals may be counterproductive and act as a deterrent to consumers seeking professional advice to maintain their right to patient autonomy (Popattia and La Caze, 2021). For reinforcement of the knowledge base about T&CM products, it is equally important for regulatory authorities to prioritize pharmacovigilance of T&CM products (also referred to as phytovigilance) and for pharmacists to report any suspected adverse reactions (Memişoğlu and Otlatıcı, 2022).

### 4.1 Strengths and limitations

This study has some limitations, some of which were foreseen as participants were all recruited online during the COVID-19 pandemic and may not be fully representative of the entire pharmacist population, and the findings cannot be extrapolated to the views of pharmacists in other countries. Willingness to participate and responses may have been impacted by the work demand and physical and mental constraints during such difficult times. Nevertheless, the response rates were collectively high, and the nine countries represented diversity in demographic and geographic considerations. Although the response rates were pleasingly high, the actual sample sizes in each country required to reach a confidence interval of 95th centile and conduct analysis for predictors were not possible, further limiting the scope and interpretation of this work. Furthermore, although a rigorous method was applied to translation from English to four other languages, nuances in the interpretation of terms and definitions used were unavoidable.

In this study, we measured support for the general concept of pharmacists’ professional responsibilities regarding T&CM products and the actions needed without an in-depth commentary or description. Although the items were informed by a systematic review of the literature and an iterative process with practicing pharmacists for both face validity and relevance, we could not verify content validity. Therefore, pharmacists’ responses may not reflect an understanding of the complexities involved in assuming such responsibilities or actions, and we may have inadvertently introduced hypothetical bias. However, the general measure of opinions may be more suitable for the purpose of this study, as this allowed synchronous reasoning across different local and national settings.

## 5 Conclusion

A set of professional practice responsibilities and stakeholder-enabling actions related to the provision of T&CM products have been proposed to pharmacists across nine countries and received varying degrees of agreement. The variations in agreements are likely associated with variations in the healthcare systems they practice and the professional education they receive. The results of this study serve as a foundation for developing T&CM product guidelines for discussions by the pharmacy profession and related stakeholders to develop guidelines. Should these professional responsibilities be enabled and actioned, there are broad-reaching and positive implications for public health and safety.

## Data Availability

The original contributions presented in the study are included in the article/Supplementary Material. Further inquiries can be directed to the corresponding author.
