# Supplementary material for: Defining and supporting a professional role for pharmacists associated with traditional and complementary medicines: a cross-country survey of pharmacists
Source: Front Pharmacol. 2023 Aug 16;14:1215475. doi: 10.3389/fphar.2023.1215475 (PMC10467277; doi:10.3389/fphar.2023.1215475)
Supplement: Supplementary file 1 [file DataSheet1.pdf]

Supplementary File S1 - Study population size and number of participants who completed the survey

Participating countries	Number of pharmacists in the country and number of pharmacists participated in this study							The role of traditional and complementaty medicine (T&CM) in the health system	The qualification system of pharmacist
	Data year	Data source	Population	Pharmacist population	Pharmacist density per 10,000 population	Minimum sample size required to achieve a 95% confidence interval, with a margin of error 5%	Number of participants who completed the survey		
Cabo Verde	2018	WHO (The state of the health workforce in the WHO African Region, 2021. Brazzaville: WHO Regional Office for Africa; 2021. Licence: CC BY-NC-SA 3.0 IGO)	571,202	172	3.01	120	61	Many people rely on plants to treat health conditions, which are commonly sold in local markets, rooted in local insular culture. The country is working on the basic law and ordinances for approval and effective implementation of T&CM in health structures, which will allow the certification of T&CM products and professionals. To improve the quality of care, a better standardization of how professional practice with T&CM and a better understanding of how people are using T&CM is urgently needed.<br><br>*Sources:*<br>*1. Romeiras MM, Catarino L, Torrão MM, Duarte MC. Diversity and origin of medicinal exotic flora in Cape Verde Islands. Plant Ecology and Evolution. 2011 Jun 15;144(2):214-25.*<br>*2. Ministry of Health and Social Security of Cape Verde, 2023.https://minsaude.gov.cv/.*	The Order of Pharmacists of Cape Verde (OFCV) regulates the qualification system for pharmacists. Upon pre-registration, pharmacists need to complete a university degree that confers the degree of pharmacist, according to the curriculum established by the Cape Verde Association of Pharmacists and recognized by the competent authorities in the country. The minimum criteria for accepting registration of pharmacists with the OFCV are based on European Directive 2013/55/EU of the European Parliament, 20/11/2013, which amends Directive 2005/33/EC. The system for revalidating professional cards based on professional development credits (CDP) is yet to be implemented.<br><br>*Sources:*<br>*The Order of Pharmacists of Cape Verde (OFCV), https://ofcv.cv/index.php/j-page/requisitos.*
China	April, 2023	Certification Center for Licensed Pharmacist of the National Medical Products Administration (http://www.cqlp.org)	1,414,417,308	735,497	5.20	384	540	In China, T&CM has been completely integrated into the health care system. Traditional Chinese medicine and conventional medicine are practiced alongside each other at every level of the health-care service. both government and commercial insurance (including both state-owned and private insurance companies) cover both traditional Chinese medicine and conventional medicine.<br><br>*Sources:*<br>*1. WHO global report on traditional and complementary medicine 2019. Geneva: World Health Organization; 2019.*<br>https://www.who.int/publications/i/item/978924151536	China has two types of pharmacist qualification systems. The first is a professional qualification system, under which only pharmacists who pass the national pharmacist licensing exam can obtain a Licensed Pharmacist certificate. People with secondary, Bachelors, or Masters degrees and the corresponding working experience requirements (depending on the academic qualification) can apply for the examination. This type of pharmacist works mainly in institutions where medicines are manufactured, distributed, or used (such as community pharmacies). The second pharmacist qualification system is a specialized system commonly seen in medical institutions, under which stringent, structured and ongoing clinical pharmacy training is provided to pharmacists. The registration of licensed pharmacist is valid for 3 years. Applicants for re-registration should meet specific requirements and provide evidence of continuing education.<br><br>Sources:<br>*1. Fang Y. Pharmaceutical policy in China. Pharmaceutical policy in countries with developing healthcare systems. 2017:169-91.*<br>2. Certification Center for Licensed Pharmacist of National Medical Products Administration, 2023. http://www.cqlp.org/
Croatia	August, 2016	European Association of Employed community Pharmacists in Europe (https://epheu.eu)	4,174,000	3,545	8.49	347	203	T&CM is not integrated into the Croatian health care system. Therefore, the insurance does not cover T&CM therapy in the country. However, some herbal products are registered on the market as over-the-counter pharmaceutical products which might be subject to at least commercial insurance.<br><br>*Sources:*<br>*1. Bukic J, Rusic D, Mas P, Karabatic D, Bozic J, Seselja Perisin A, Leskur D, Krnic D, Tomic S, Modun D. Analysis of spontaneous reporting of suspected adverse drug reactions for non-analgesic over-the-counter drugs from 2008 to 2017. BMC Pharmacol Toxicol. 2019 Oct 18;20(1):60. doi: 10.1186/s40360-019-0338-2.*	Every pharmacy student who finishes pharmacy study and obtains masters degree in Croatia has the opportunity to get licensed by Croatian Chamber of Pharmacist, without the need to pass any additional exams. There is only Master degree education in the field of pharmacy available in Croatia, and not the Bachelor degree. Members of Croatian Chamber of Pharmacists are obligated to participate in continuing education and obtain 30 points in 6 years since their license is renewed after this period.<br><br>*Sources:*<br>*1. Croatian Chamber of Pharmacy, 2023. https://www.hljk.hr/about-the-chamber-a41*
Lebanon	2018	The Lebanese Order of Pharmacists (http://opl.org.lb)	4,355,913	8,855	20.33	369	627	In Lebanon, both traditional Lebanese medicine and conventional medicine coexist. Individuals have the freedom to choose the approach to healthcare that aligns with their beliefs and preferences. It is common for individuals to seek a balance between the two systems, utilizing traditional remedies for certain conditions while relying on conventional medicine for more complex or acute health issues.<br><br>*Sources:*<br>*1. Naja F, Alameddine M, Itani L, Shoaib H, Hariri D, Talhouk S. The use of complementary and alternative medicine among lebanese adults: results from a national survey. Evidence-Based Complementary and Alternative Medicine. 2015 Jan 1;2015*	In Lebanon, the qualification system for pharmacists involves both pre-registration education and post-registration requirements. With the pre-registration education, the first step to becoming a pharmacist in Lebanon is to complete a five-year Bachelor of Pharmacy (B.Pharm) program at an accredited university. During the B.Pharm program, pharmacists are required to undergo a 12-month internship in a community or hospital pharmacy under the supervision of a licensed pharmacist. Following the internship, individuals must pass the National Pharmacy Exam conducted by the Lebanese Ministry of Public Health. Upon successfully passing the National Pharmacy Exam, candidates can apply for a license from the regulatory body, the Lebanese Order of Pharmacists (LOP). Pharmacists who are registered with the LOP are required to do a 30 CE per two years for their license to remain active.<br><br>*Sources:*<br>*1. Lebanese Order of Pharmacists, 2023. https://www.opl.org.lb/*

Malaysia	September, 2016	Malaysian Pharmacists Society (https://www.mps.org.my/)	31,530,000	12,294	3.90	373	379	The T&CM practices in Malaysia exhibit a multiethnic and multicultural nature, wherein a diverse range of T&CM modalities coexist. The Ministry of Health (MoH), Malaysia has officially recognized seven distinct forms of T&CM namely Traditional Malay Medicine (TMM), Traditional Chinese Medicine (TCM), Traditional Indian Medicine (TIM), Homeopathy, Chiropractic, Osteopathy, and Islamic Medical Practice (IMP). Over the past two decades, the Malaysian government has developed policies to institutionalize and integrate those T&CM into the national health care system. Additionally, numerous herbal medicine and dietary supplement products are sold over the counter and being used by consumers as T&CM.<br><br>Sources:<br>*1. Ministry of Health Malaysia. Traditional and complementary medicine blueprint health care 2018–2027. 2018. https://hq.moh.gov.my/tcm/ms/upload/TCMBlueprint_HealthCare.pdf*	To become a registered pharmacist in Malaysia, individuals must possess a pharmacy degree that has been duly recognized by the Pharmacy Board of Malaysia. Upon completion of the pharmacy degree, individuals are required to pass the Qualifying Examination to practice pharmacy in Malaysia. This examination is a mandatory requirement for individuals seeking registration with the Pharmacy Board of Malaysia. In addition to passing the Qualifying Examination, individuals who have graduated from pharmacy programs must complete a one-year training program as Provisionally Registered Pharmacists (PRP) at recognized facilities before being eligible to apply for full registration as a Fully Registered Pharmacist (FRP). In order to obtain the Annual Retention Certificate (ARC), a minimum of 30 CPD points collected within. 2 years are required for ARC application and renewal.<br><br>*Sources:*<br>*1. Wahab MS, Ali AA, Zulkifly HH. Liberalisation of pharmacist training: the need to reflect on pharmaceutical education in Malaysia. Journal of Pharmacy Practice and Research. 2013;43(2):162.*<br>*2. Pharmaceutical Services Programme, Ministry of Health Malaysia, 2023. https://www.pharmacy.gov.my/v2/en*
Philippines	October, 2021	Human Resources for Health (HRH) Statistics, Department of Health (https://bit.ly/doh-hrhstat)	110,198,654	5,167	0.47	358	196	The Philippines first legally recognized the value of T&CM in 1997 with the Traditional and Alternative Medicine Act (TAMA Law), which regulates its practice, research, and related medicines. However, despite two decades of legislation, progress toward integrative healthcare remains sluggish, partly from the deep-seated divide between biomedical and T&CM practice. Patients with poor economic backgrounds face geographic obstacles to healthcare, unaffordable conventional therapies, and underdeveloped palliative and survivorship care. These barriers, compounded by low socioeconomic status and poor health literacy, drive patients to seek alternative forms of treatment, such as T&CM.<br><br>*Sources:*<br>*1. Palileo-Villanueva LM, Palafox B, Amit AM, Pepito VC, Ab-Majid F, Ariffin F, Balabanova D, Isa MR, Mat-Nasir N, My M, Renedo A. Prevalence, determinants and outcomes of traditional, complementary and alternative medicine use for hypertension among low-income households in Malaysia and the Philippines. BMC complementary medicine and therapies. 2022 Sep 30;22(1):252.*	A registered pharmacist in the Philippines must go through rigorous education and training before practicing pharmacy. Typically, pharmacy is a four-year course program in most colleges and universities. However, some universities offer five-year pharmacy programs. Upon graduation, pharmacy graduates are required to undergo 360 hours of major post-graduate pharmacy internship. This requirement is set on top of the 600 total hours (200 hours each in hospital, manufacturing, and community pharmacy) of minor internships taken by undergraduate students. Pharmacy graduates are required by law (Republic Act 10918: Philippine Pharmacy Act) to undergo the Pharmacists Licensure Examination (PhLE) conducted by the Professional Regulation Commission (PRC) to become registered pharmacists and practice pharmacy in the Philippines. A licensed pharmacist is required to complete 45 CPD credit units every renewal. The TAMA Law of 1997 states the creation of the Philippine Institute of Traditional and Alternative Health Care under the Department of Health which organizes and develops continuing training programs for physicians, nurses, pharmacists, physical therapists, and other professional health workers and students, as well as scientists, research managers and extension workers in the field of traditional and alternative health care; TAMA Law and T and CM has been integrated as part of the curriculum in the undergraduate and post-graduate degrees for Pharmacist under CAM subject.<br><br>*Sources:*<br>*1. The Professional Regulation Commission (PRC), 2023. https://prc.gov.ph/pharmacy*
Portugal	2021	Statistics Portugal (https://www.ine.pt/xportal/xmain?xpid=INE&xpgid=ine_main&xlang=pt)	10,421,117	16,055	15.41	376	82	T&CM in the Portuguese healthcare system enjoys a tolerant status, with legally recognised practitioners practising outside the national system. The seven state-regulated non-conventional therapies include traditional Chinese medicine, homoeopathy, and herbal medicine. These practitioners, accredited upon completion of a degree certified by a health or higher education institution, prescribe different therapeutic regulated products, such as drugs and medicines covered by the national Medicines Statute, thus complying with EU Directives and guidelines. For instance, the legal regime for homoeopathic and herbal medicines includes manufacture, import and export, marketing, advertising, pharmacovigilance, and dispensing for human consumption through community pharmacies<br><br>*Sources:*<br>*1. WHO global report on traditional and complementary medicine 2019. Geneva: World Health Organization; 2019. https://www.who.int/publications/i/item/978924151536*	The Portuguese Pharmaceutical Society (Ordem dos Farmacêuticos, OF) regulates the Portuguese qualification system for pharmacists. Pre-registration, pharmacists needed to complete a 5-years pharmaceutical master's degree obtained at a nationally accredited higher education institution. All public and private accredited university graduates are automatically issued a professional license to practice. Post-registration, pharmacists must gain 15 continuous professional development credits from OF-accredited courses and professional activities every five years to keep their license to practice. To become a pharmacist specialist (e.g. community, hospital, industry, etc.), the pharmacist must practice for several years in that area and undergo an examination by the respective speciality college. Mandatory Continuing Professional Development (CPD) programs have been established by a decree/governmental regulation and a code/regulation by professional body in Portugal. The compliance with mandatory CPD requirements is taken into account in the review of the licence to practise.<br><br>*Sources:*<br>*1. Portuguese Pharmaceutical Society (Ordem dos Farmacêuticos, OF), 2023. https://www.ordemfarmaceuticos.pt/pt/*<br>*2. CED, CPME, EFN, EMA, EPHA, PGEU. Final report—EAHC/2013/Health/07 - Study concerning the review and mapping of continuous professional development and lifelong learning for health professionals in the EU; 2013*

Saudi Arabia	2021	Ministry of Health (https://www.moh.gov.sa/)	34,110,821	30,840	9.04	380	71	In Saudi Arabia, diverse traditional healing practices from various geographical and cultural origins exist. The National Center for Complementary and Alternative Medicine (NCCAM) that was founded by the Ministry of Health, serves as Saudi Arabia's national reference for T&CM related activities. T&CM providers offer their services in public and private hospitals and clinics, and their practice requires a license issued by the authorities. Religious healings, herbal medicine, cupping therapy, healing with honey, Camel milk and cautery are common traditional therapies in this country.<br><br>*Sources:*<br>*1. WHO global report on traditional and complementary medicine 2019. Geneva: World Health Organization; 2019. https://www.who.int/publications/i/item/978924151536.*<br>*2. Mohamed K.M. Khalil, Sulaiman Al-Eidi, Meshary Al-Qaed, Saud AlSanad, The future of integrative health and medicine in Saudi Arabia, Integrative Medicine Research, Volume 7, Issue 4, 2018, Pages 316-321.*	In Saudi Arabia, the qualification system of pharmacists has three levels in sequence: a pharmacist, a pharmacy specialist, and a pharmacy consultant. All those qualifications are obtained through the Saudi Commission for Health Specialties (SCFHS) which is the pharmacy regulation commission. Pre-registration in the SCFHS as a pharmacist, all applicants must have a bachelor-degree in pharmaceutical sciences or its equivalent (Doctor of Pharmacy degree) either from a national institute or an accredited international institute. After sitting for the examination and pass, they obtain a pharmacist license (level 1). Other levels of qualifications can be obtained by finishing a master or a PhD degree (level 2 & 3, respectively). Also, pharmacists completing a residency and/or fellowship training in the field of pharmacy could advance to level 2 & 3. Furthermore, all pharmacy jobs at any given sector such as academia, hospitals, community pharmacies and pharmaceutical manufacturing…etc. must have a pharmacist license to practice in Saudi Arabia. To renew the license, pharmacists need to have at least 40 hours of CME every 2 years.<br><br>*Sources:*<br>*1. Saudi Commission for Health Specialties (SCFHS), 2023. https://www.scfhs.org.sa/en*
Thailand	2021	National Statistical Office (http://www.nso.go.th/sites/2014en/Pages/default.aspx)	66,090,475	16,090	2.43	376	182	In Thailand, the policy on T&CM is integrated into the National Health Act (B.E.2550) of 2007. There is also a national policy exclusively on traditional Thai medicine (TTM), which is included in the10th National Health Development Plan, 2007–2011. Legislation related to T&CM includes laws and regulations on the “practice of the art of healing” (B.E.2542), pharmaceuticals (B.E.2510) and the protection and promotion of TTM (B.E.2542). T&CM products cover a variety of products ranging from vitamins and minerals to herbal products in Thailand. Regulation of T&CM products in Thailand depends on the character of the product and usually are regulated by either the Food Control Division (Food Act, 1979) or Herbal Product Division (Herbal Product Act, 2019). Although T&CM products cannot make health claims, they can distribute to broader channels and can make direct to consumer advertisements. Obviously, T&CM products are part of the healthcare system. People use them for self-care and must pay out-of-pocket as they are not included in the pharmacy benefits package.<br><br>Sources:<br>1. WHO global report on traditional and complementary medicine 2019. Geneva: World Health Organization; 2019. https://www.who.int/publications/i/item/978924151536	In Thailand, there are 19 faculties of Pharmaceutical Sciences (including 14 public and 5 private faculties), all of which offer a 5-year Bachelor of Science degree in Pharmacy curriculum. All pharmacy graduates automatically became registered pharmacists with lifelong pharmacist licenses. Until 2005, the BSc. curriculum was discontinued and the 6-year Pharm D curriculum was inaugurated. Pharm D graduates must take the pharmacy license examination. Pharmacist license is valid for 5 years. All pharmacists, both BSc and Pharm D graduated must re-license by participating in the continuing education for 100 credits in 5 years with minimum of 10 credit earned per year. As of now, the pharmacy license is not differentiated by pharmacist's specialties, but it is likely that the license will be diversely classified e.g. patient oriented, product oriented, consumer protection oriented.<br><br>Sources:<br>1. The Pharmacy Council of Thailand, 2023. https://www.pharmacycouncil.org/index.php#gsc.tab=0
